# In situ labeling of non-accommodating interneurons based on metabolic rates

**DOI:** 10.1016/j.redox.2020.101798

**Published:** 2020-11-28

**Authors:** G.C. Gotti, M. Kikhia, V. Wuntke, L.A. Hasam-Henderson, B. Wu, J.R.P. Geiger, R. Kovacs

**Affiliations:** a Institut für Neurophysiologie, Charité – Universitätsmedizin Berlin, Corporate Member of Freie Universität Berlin, Humboldt-Universität zu Berlin, Berlin Institute of Health, NeuroCure Cluster of Excellence, Berlin, Charité Platz 1, 10117, Berlin, Germany; b Institute of Neuroinformatics, University of Zurich – Irchel, Winterthurerstrasse 190, 8057, Zürich, Switzerland

**Keywords:** 2',7'-Dichlorodihydrofluorescein, Fast spiking interneuron, Neurometabolic coupling, VGAT-YFP, Seizure, Energy metabolism

## Abstract

Maintaining high frequency firing of narrow action potentials puts a large metabolic load on fast spiking (FS), perisomatic-inhibitory interneurons compared to their slow-spiking, dendrite targeting counterparts. Although the relationship of action potential (AP) firing and metabolism is firmly established, there is no single method to differentiate interneurons *in situ* based on their firing properties.

In this study, we explore a novel strategy to easily identify the metabolically active FS cells among different classes of interneurons.

We found that the oxidation of the fluorescent free radical marker 2,7-dichlorodihydrofluorescein (H_2_DCF) preferentially occurs in interneurons both in slice cultures and acute brain slices. Despite their morphological heterogeneity, almost all DCF-positive (DCF+) neurons belonged to the cluster of non-accommodating FS interneurons. Furthermore, all FS interneurons expressing parvalbumin (PV) both in slice cultures and in acute slices from tdTomato-PVCre transgenic mice were also DCF+. However, only half of the recorded DCF + cells were also PV+, indicating that H_2_DCF-oxidation occurs in different interneuron classes characterized by non-accomodating AP-firing. Comprehensively enhancing spontaneous neuronal activity led to mitochondrial oxidation of DCF in pyramidal cells as well as interneurons, suggesting that the apparent selectivity towards interneurons represents differences in the underlying metabolic load.

While radical-scavenging, inhibition of APs or NO-synthesis, and iron chelation had no effect on the staining pattern, exposure to the complex-I inhibitor, rotenone, prevented interneuronal DCF accumulation. We conclude that H_2_DCF oxidation is independent of free radicals but correlates with the intensive oxidative energy metabolism and high mitochondrial mass in interneurons sharing the non-accommodating FS phenotype.

## Highlights

• Fast spiking non-adapting interneurons preferentially oxidize and accumulate H_2_DCF.
• Labeling of interneurons correlates with mitochondrial mass and energy metabolism.
• Free radical formation does not contribute to DCF-labeling of interneurons.

## Graphical abstract

**Figure:**
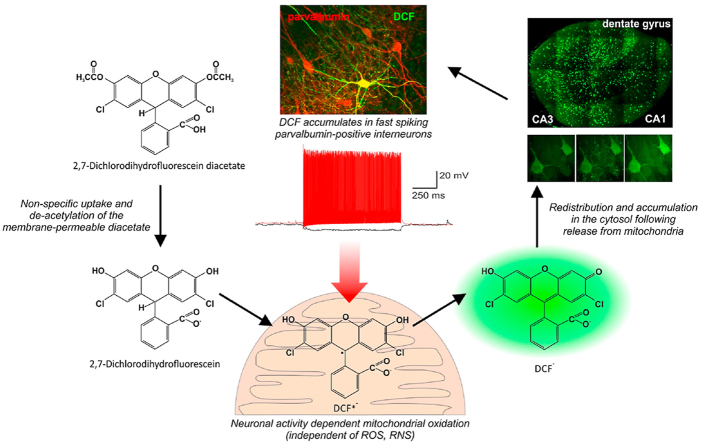

## 1 Introduction

Differences in the active electrophysiological properties of pyramidal cells and fast or slow spiking interneurons are reflected in their metabolic load and mitochondrial capacity [[1], [2], [3], [4]]. Maintaining the high frequency firing of narrow action potentials represent a large load to energy metabolism [5,6] and is associated with high mitochondrial mass, as was shown in the parvalbumin (PV) containing class of fast-spiking (FS) interneurons [7]. Recent findings suggest that this relation is not restricted to PV positive cells. Deep sequencing based classification of CA1 interneurons indicated the presence of a ‘latent factor' related to spiking properties and energy metabolism across different classes of interneurons [8]. The expression of metabolic enzymes and ion channels with fast gating properties varied between soma-targeting and distal dendrite-targeting interneurons, supporting the role of these ion channels in shaping energy metabolism. While the classification of interneurons relies on the cell type-specific expression of calcium binding proteins, neuropeptides, and transmitter receptors [8,9], there is currently no known common indicator of the fast-spiking phenotype across different interneuron classes *in situ.*

In the course of oxidative phosphorylation, a small percentage of oxygen is incompletely reduced to superoxide anion. Therefore, the higher metabolic load of FS interneurons might come at the cost of increased reactive oxygen species (ROS) formation. Indeed, in a previous study using the fluorescent free radical marker 2′,7′-dichlorodihydrofluorescein (H_2_DCF) in organotypic hippocampal slice cultures, we discovered intensive labeling of the mitochondrial network in some neurons. These cells were identified as interneurons based on morphological criteria [10]. However, we were unable to identify a single morphological marker which would predict whether a cell will accumulate DCF, nor were we able to prove that DCF accumulation correlates with enhanced ROS formation.

Although H_2_DCF and its carboxymethyl derivative (CM-H_2_DCF) have been widely used as biomarkers for ROS formation and oxidative stress in general [11], many studies have confirmed that H_2_DCF oxidation is a nonspecific reaction that does not solely depend on peroxide or other ROS [12]. The oxidation of H_2_DCF by peroxide is slow but can be accelerated by the presence of catalysts such as peroxidases or ferrous iron via Fenton-type reactions [[13], [14], [15]]. Thus, cytosolic accumulation of fluorescent DCF could also be an indicator of an increase in the catalyst such as cytochrome-c [15] or lysosomal iron [16] rather than peroxide formation *per se*. Additionally, thiol radicals or reactive sulfide species were also suggested as putative oxidants for H_2_DCF [17], making the interpretation of DCF fluorescence contentious. High oxidative capacity and mitochondrial mass would also represent higher levels of potential oxidants, such as labile ferrous iron, cytochrome-c or peroxide.

In this study, we explored the conspicuously unequal oxidation of DCF by different neurons and aimed to discover the mechanisms underlying the irregular fluorescence pattern. We obtained whole cell patch clamp recordings of DCF positive (DCF+) cells in slice cultures from wild type (WT) Wistar rats and compared their electrophysiological properties to interneurons carrying Venus yellow fluorescent protein on the vesicular GABA transporter (VGAT-YFP). To exclude an interference of the culturing process on DCF-labeling, we applied the fluorescent marker to acute slices from Wistar rats as well as from transgenic mice carrying tdTomato in PV + cells. We determined the spatio-temporal pattern of H_2_DCF oxidation during 4-aminopyridine (4AP) induced synchronous neuronal activity. To identify the process underlying H_2_DCF oxidation, we repeated the staining procedure in the presence of (1) the mitochondrial superoxide-scavenger MitoTEMPO, (2) the electron transport chain complex-I inhibitor rotenone, (3) the NO-synthase inhibitor 7-nitroindazole (7NI), (4) the iron chelator Clioquinol, and (5) the sodium channel inhibitor tetrodotoxin (TTX).

## 2 Methods

For a detailed description of the experimental procedures please refer to “Supplementary Data - Methods”.

### 2.1 Preparation of acute brain slices and organotypic hippocampal slice cultures

Animal care and handling was in accordance with the WMA Declaration of Helsinki and institutional guidelines (https://experimentelle-medizin.charite.de/). Protocols for organ removal and culturing were approved by the State Office of Health and Social Affairs Berlin (https://www.berlin.de/lageso/) under the license numbers: T0123/11 and T0045/15. Organotypic hippocampal slice cultures were prepared and maintained according to the Stoppini method from both wild type (WT) and VGAT-YFP Wistar rats of either sex [18,19]. Acute hippocampal brain slices were prepared from WT Wistar rats as well as from tdTomato parv-Cre transgenic mice that express a red fluorophore in PV + neurons [20,21]. Slice cultures were used for experiments between 5 and 14 days in vitro (DIV).

### 2.2 Electrophysiology

Acute rat brain slices or slice cultures were transferred to a recording chamber perfused with warm (30–32 °C), carbogen saturated (95% CO_2_, 5% O_2_) artificial cerebrospinal fluid (aCSF, in mM: NaCl 129, KCl 3, NaH_2_PO_4_ 1.25, MgSO_4_ 1.8, CaCl_2_ 1.6, NaHCO_3_ 26, and glucose 10, pH 7.3). Whole cell recordings were obtained from fluorescent cells in VGAT-YFP or DCF-stained WT slice cultures (with pipette solution containing K-gluconate, 135, HEPES 10, KCL 6, MgCl_2_ 2, EGTA 0.2, Na_2_ATP 2, Na_2_GTP 0.5, Na_2_-Phosphocreatine 5, Biocytin 0.1%, or K-gluconate 135; NaCl 4; CaCl_2_ 0,05; HEPES 10; EGTA 1; Mg-ATP 2, 270–290 mOsm, pH 7.3) by using MultiClamp 700B (Axon CNS, Molecular Devices, Sunnyvale, CA, USA) or Pico2 (Tecella, Foothill Ranch, CA, USA) amplifiers. Zero net current membrane potential (Em), membrane capacity (Cm), membrane resistance (Rm) and access resistance (Ra), were determined automatically in voltage clamp (VC) immediately after breakthrough. In current clamp mode (CC) bridge balance was automatically compensated before recording 1 s long hyperpolarizing and depolarizing steps of 25 mV to evoke AP-trains.

### 2.3 Immunohistochemistry

Following electrophysiological recording, slice cultures were processed for immunofluorescence labeling of parvalbumin (mouse anti-parvalbumin antibody 1:1000; Millipore, goat anti-mouse Cy3 secondary antibody (1:100; Millipore)) and DCF + cells filled with 0,1% Biocytin during electrophysiology were visualized using avidin-conjugated Alexa Fluor488.

### 2.4 Pharmacology

Epileptiform activity was induced by adding 4AP (4-AP, 100 μM, Bio-Techne GmbH, Wiesbaden-Nordenstadt, Germany) to the aCSF [18,22]. To narrow down the underlying cause of H_2_DCF oxidation, slice cultures were treated with the following substances prior to and during DCF-staining: the mitochondrial superoxide-scavenger (2-(2,2,6,6-tetramethylpiperidin-1-oxyl-4-ylamino) -2-oxoethyl) triphenylphosphonium chloride (Mito-TEMPO, Santa Cruz Biotechnology, Inc, Dallas, TX, USA); the electron transport chain complex I inhibitor rotenone (Sigma-Aldrich, St. Louis, MO, USA); the neuronal NO-synthase inhibitor 7NI (Sigma-Aldrich, St. Louis, MO, USA); the iron chelator 5-Chloro-7-iodo-8-quinolinol (Clioquinol, BioVision, Milpitas, CA, USA) as well as the voltage gated sodium channel inhibitor tetrodotoxin (TTX, BIOTREND Chemicals AG, Cologne, Germany) and the glutamate receptor inhibitors 6-cyano-7-nitroquinoxaline-2,3-dione (CNQX), (2R)-amino-5-phosphonopentanoate (DL-APV, 50 μM each) (Table 1).

**Table 1:** Treatment protocols.

Group	Number of cultures	Treatment duration	Drug concentration	Staining duration
Control	35	NA	NA	30–60 min
Mito-TEMPO	16	overnight	200 μM	60–90 min
7-NI	17	120–150 min	200 μM	60–90 min
Rotenone	17	30–60 min	5 μM	30–60 min
Clioquinol	17	90–120 min	50 μM	30–60 min
TTX (CNQX, DL-APV)	18	68–74 h	1 μM	30–60 min

### 2.5 Image acquisition and evaluation

Fluorescence recordings of the kinetics and pharmacology of H_2_DCF oxidation were obtained with a spinning disk confocal microscope (Andor Revolution, BFIOptilas GmbH, Gröbenzell, Germany). Slice cultures were incubated prior to the image acquisition with the membrane permeable probes H_2_DCF-DA or CM-H_2_DCF-DA (20 μM), for kinetics and pharmacology, respectively. The slices from an individual culture membrane were imaged one after another, resulting in an approximately 30-min delay between slices. Following staining in the presence of the aforementioned drugs, 8–15 Z-stacks were obtained covering all the regions of the hippocampus. From each field of view, ROIs of arbitrarily selected cells and parenchymal regions were used to examine cytosolic DCF accumulation relative to background. While the absolute fluorescence depended on the duration of the staining, hence on the rank of a slice from a culture membrane, the relative fluorescence (cell vs parenchym) showed no significant differences between first and second slice within an individual treatment group (relative fluorescence depending on duration of the staining, p: 0.55, 0.128, 0.55 for DG, CA3, CA1)).

To measure the activity-dependent oxidation of the probe, z-stacks were obtained over the CA3 while simultaneously recording the local field potentials of 4AP-induced network activity. Exposure (100 ms) and sampling frequency (every 20–30 s) were kept low to minimize the light-induced auto-oxidation of the probe. Putative mitochondrial contribution to the cytosolic DCF fluorescence was isolated by using spatial frequency filtering as described in [23], while presumably unrelated fluorescence changes were measured over cytosolic regions apparently free of mitochondria (nucleus). Changes in the DCF fluorescence intensity are presented as Δf/f_0_ over time, f_0_ representing the average fluorescence of the first 1 min of the recording.

DCF and PV positivity was quantified in panorama images of the entire hippocampal slice rendered by using the large scan function provided with Nikon's A1R multiphoton confocal microscope (25 × N.A. 1.1 objective, Nikon, Shinagawa, Tokyo, Japan) at the AMBIO Life Cell Imaging Core Facility (AMBIO.charite.de). After imaging a vital DCF-stained slice, the same function was used to obtain a congruent picture after immunohistochemistry for PV (InSight DeepSee pulsed TiSa laser (Spectra-Physics, Santa Clara, CA, US), 960 nm and 1040 nm laser lines for DCF and Cy3, respectively). To overcome the inevitable shrinkage and distortion of the fixed tissue and obtain superimposable reconstructions, we used a hallmark based image registration procedure (ImageJ plugin BigWarp [24]) and the CellCounter plugin to determine the percentage of DCF + PV + cells.

### 2.6 Statistics

Statistical analysis was conducted in the SPSS software package (IBM, SPSS 24). Electrophysiology recordings from 122 VGAT-YFP and 83 WT cultures were used for statistical analysis (DIV evenly distributed between groups, Kruskal-Wallis Test: H(1) = 2,622, p = 0,105, Levene statistic: 0,884, p = 0,348). Following individual distribution tests and comparison of the medians of VGAT-YFP and WT electrophysiological variables (Shapiro-Wilk test, histograms, Q-Q Plots, Mood's median test), we used binary logistic regression to investigate the relationship between the categories of *firing pattern* (dependent variable) and predictor variables (including DCF and electrophysiological data). For the list of variables, further details on the model procedure, and the selection process of explanatory variables please refer to the supplemental statistics table and description. Because the data on DCF accumulation obtained in different treatment groups was not normally distributed, we used the nonparametric median test with a pairwise post-hoc test to compare the effects of different treatments on the oxidation of H_2_DCF, with significance values adjusted by the Bonferroni correction for multiple tests. The nonparametric Friedman test was used to compare the relative fluorescence values between the three hippocampal areas i.e. dentate gyrus (DG), CA1, and CA3 in the control group. For pairwise comparison, Wilcoxon signed-rank test and Sign tests were used with Bonferroni adjustment. A Wilcoxon signed-rank test was also used to compare the rise in the fluorescent signal within 1 min before and during seizures.

## 3 Results

### 3.1 Morphological and electrophysiological properties of DCF + interneurons

**Fig. 1:**
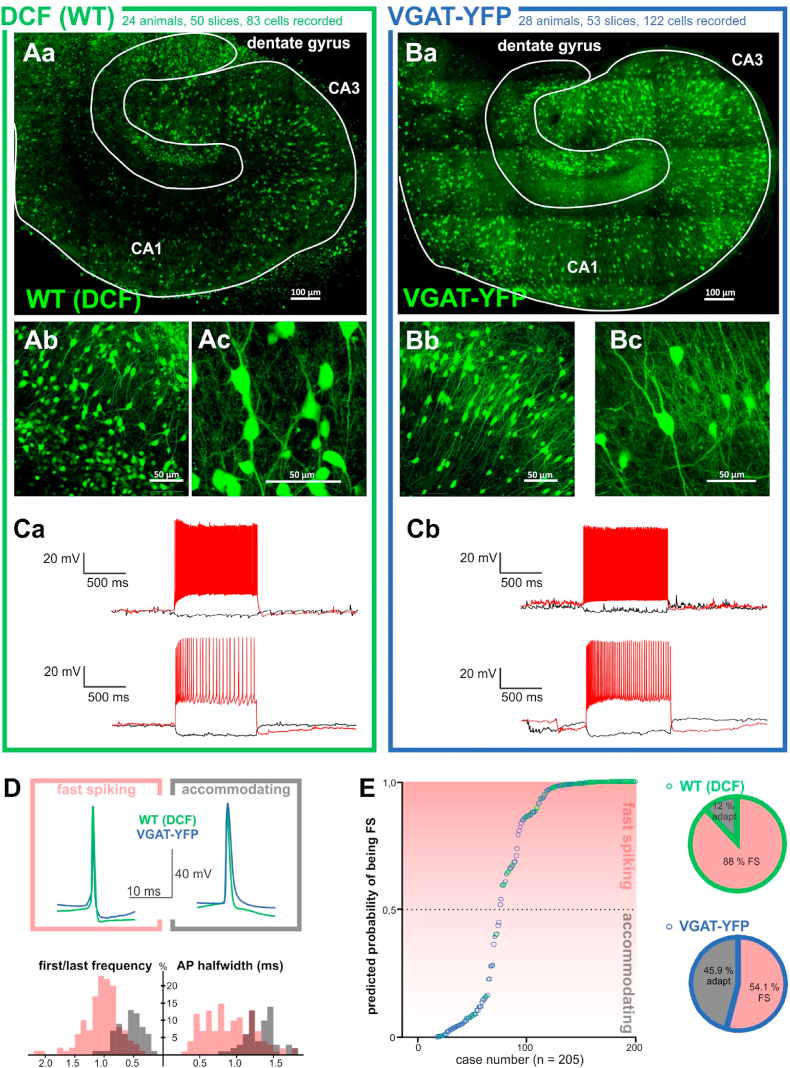
Comparison of DCF positive cells and interneurons in the hippocampus (A - C. Green and blue borders outline results from DCF + WT (left) and VGAT-YFP (right) organotypic hippocampal slice cultures.) A. DCF fluorescence pattern in a hippocampal slice culture from a wild type (WT) wistar rat at different magnifications. (Aa.) reconstruction of the whole hippocampus with individual regions delineated (Ab.) CA3 stratum pyramidale and (Ac.) close up of individual cells displaying the morphological characteristics of interneurons. B. Distribution of fluorescent interneurons in slice culture from VGAT-YFP rats. (Ba.) reconstruction of the whole hippocampus (Bb.) CA3 stratum pyramidale (Bc.), close up of individual cells in the CA3. C. Exemplary traces of fast-spiking non-accommodating and accommodating firing patterns upon a 1 s depolarization step from (Ca.) WT DCF positive cells and (Cb.) VGAT-YFP interneurons. D. Exemplary AP traces from DCF + WT (green) and VGAT-YFP (blue) cultures with comparison of non-accomodating (pale red outline, left) and accommodating (grey outline, right) interneurons as classified by binary logistic regression. E. Distribution of the two firing patterns among VGAT-YFP and DCF + cells. While both phenotypes were equally represented in YFP-interneurons, 88% of the DCF + cells were predicted to be fast-spiking, non-accommodating by logistic regression analysis. (For interpretation of the references to colour in this figure legend, the reader is referred to the Web version of this article.)

Bulk loading of hippocampal slice cultures with the diacetate ester of H_2_DCF (or its chloromethyl derivative, CM-H_2_DCF) resulted in a heterogeneous staining pattern as previously described [10]. While DCF-labeled microglial cells could be observed on the culture surface, the fluorescence below the upper ~20 μm was clearly of neuronal origin (Fig. 1A). In general, pyramidal cells of the cornu ammonis accumulated less DCF than cells that resembled interneurons in direct comparison with slice cultures from VGAT-YFP rats (Fig. 1A and B). In a few cultures, the granule cells of the DG could also accumulate more DCF than pyramidal cells, but the fluorescence remained less intense when compared to putative interneurons. Prolonging the dye exposure from 30 min to an hour resulted in a significant increase in the overall fluorescence without affecting the staining pattern (see methods).

We performed whole cell patch-clamp recordings of DCF + cells in the CA3, DG and hilar regions of slice cultures and acute slices. Based on their electrophysiological properties, none of the recorded DCF + cells was a pyramidal cell. The heterogeneity of CA3/DG interneurons, with delicate differences in dendritic and axonal arborization patterns, synaptic connectivity and firing properties, is maintained in slice cultures [18]. Irrespective of differences in morphology and location, we divided the recorded interneurons based on their firing pattern into accommodating slow spiking and non-accommodating fast-spiking (FS) interneurons [18].

We proceeded to compare the electrophysiological properties of the DCF + neurons with interneurons in age-matched slice cultures from VGAT-YFP rats (Fig. 1C–E). While the VGAT-YFP cultures had interneurons of both accommodating and non-accommodating categories, we found that DCF + cells represented a surprisingly homogenous group with electrophysiological properties typical of FS interneurons (Fig. 1CD). In order to determine the predictive force of DCF labeling for the firing pattern (FS or accommodating), we applied binary logistic regression to suitable predictor variables (see supplemental methods). We found that the non-accommodating FS category is associated with a short action potential half width, lower membrane capacity, higher access resistance, DCF positivity, and larger ratio of inter-event intervals (IEI) (computed by dividing the mean IEI of the first five by the last five action potentials evoked by a 1 s depolarization). These variables improved the prediction of a cell belonging to the FS group from 66,8% correct in the baseline model without predictor variables to 92,0% correct in the final model. While the recorded YFP + cells were fairly evenly distributed among the accommodating (45.9%) and non-accomodating FS (54.1%) categories, the majority of DCF + cells were FS and only 12% were predicted to be accommodating or intermediate forms (Fig. 1E). Together, these results compounded our observation that DCF preferentially stains interneurons of the non-accommodating FS phenotype.

**Fig. 2:**
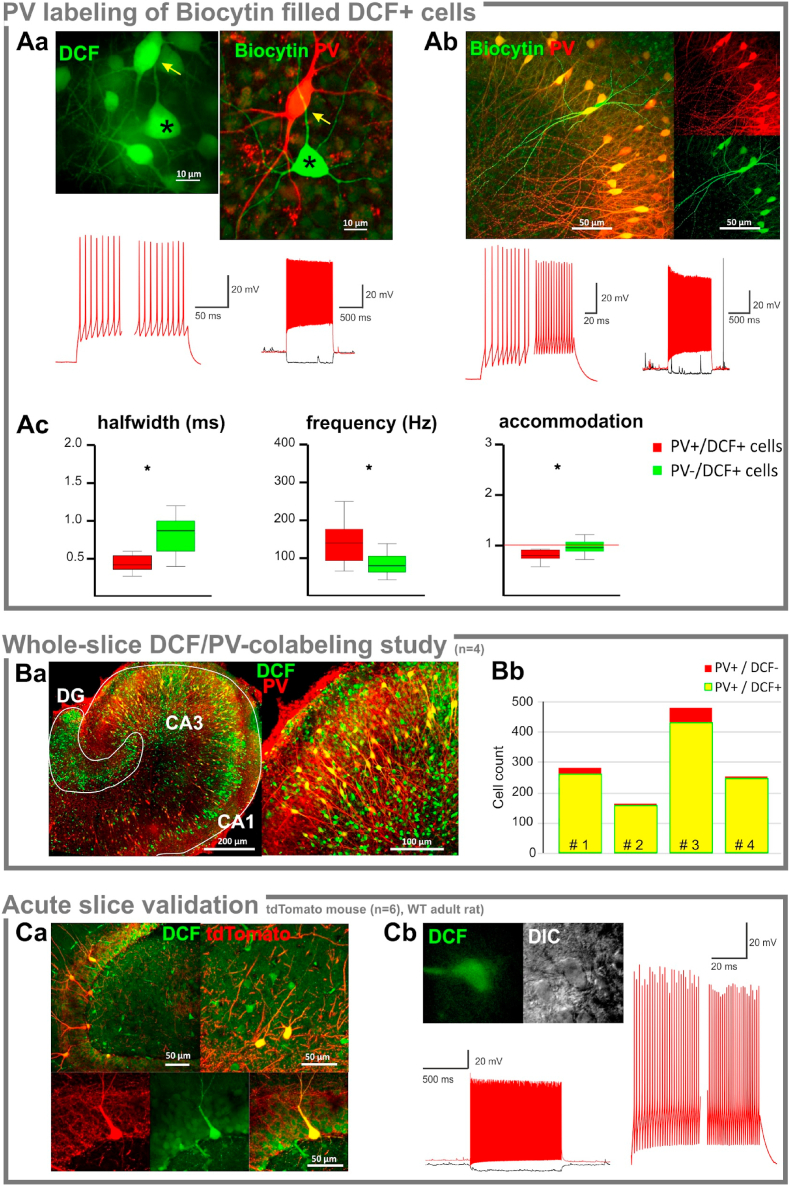
**Parvalbumin expression in DCF** + **cells** A. Parvalbumin expression was identified by immunofluorescence (red) in biocytin-filled (green) DCF + neurons following whole cell recordings. (Aa.) DCF + cells in the CA3 region of a slice culture. The recorded Biocytin-filled cell is marked by an asterisk. The image on the left represents the DCF labeling used to select a cell for recording. The picture on the right shows the same slice and region after immunohistochemistry for PV. The Biocytin-filled recorded cell is PV -. Noticeably, its neighbour (yellow arrow, no electrophysiology) is both DCF + and PV +. The electrophysiology traces on the left show a magnification of the first and last 100 ms of an AP trace upon a 1 s depolarizing pulse. Note the absence of accommodation. (Ab.) Another example of immunohistochemistry and firing pattern of a DCF + cell upon a 1 s depolarizing pulse with comparison of the first and last 100 ms showing spike frequency facilitation. While the recorded cell was both PV and DCF +, the surrounding PV + cells also present a faint CM-DCF fluorescence (Ac.) 53% of the recorded DCF + cells were also PV +. While nearly all cells were non-accommodating interneurons, the PV + cells had significantly narrower APs (*p* = 0.004), higher maximum firing rates (*p* = 0.027), and displayed frequency facilitation (*p* = 0.045, Mann-Whitney *U* test). Ba. Overlay of *in situ* DCF fluorescence and immunofluorescent PV-labeling of the same slice culture (left) and a close up of the CA3 region (right). (Bb.) Interneuron counting following the full reconstruction of four slice cultures revealed that 96% of the PV + FS interneurons were also DCF +. Ca. DCF staining (green) in acute slices from tdTomato parv-Cre transgenic mice. PV + fast spiking cells (red) were also DCF +, but not all DCF + cells were PV + (the images show excerpts from the DG, hilus and CA3) Cb. In acute slices from young adult Wistar rats, DCF accumulated exclusively in interneurons consisting of fast-spiking non-accommodating and intermediate accommodating neurons. The insert shows the DIC and fluorescent image of the recorded cell, while the traces demonstrate the fast spiking pattern and frequency facilitation between the first and last 100 ms of the depolarization step. (For interpretation of the references to colour in this figure legend, the reader is referred to the Web version of this article.)

As a subset of interneurons with FS phenotype also express the Ca^2+^-buffer protein, parvalbumin (PV) [25], biocytin-filled DCF + interneurons were processed for immunofluorescent detection of PV. We found that 53% of the recorded cells (15 out of 28 cells) were also PV+ (Fig. 2Aa-b). While all DCF + cells were subjectively deemed non-accommodating, the PV + subset of cells had narrower APs, higher maximum frequencies and showed a significant spike facilitation as compared to PV negative cells (Fig 2Ac, *p* = 0.004, 0.027, 0.045, respectively), indicating heterogeneity within the DCF + interneuron group. Indeed, biocytin-filled DCF + cells fall into several morphological categories, i.e. relatively large basket or chandelier cells in the pyramidal layer, OLM-like cells in the stratum oriens along the cornu ammonis and bitufted cells in CA3 and CA1 (Fig. 2AB).

By using the chloromethyl derivative of DCF, which had a better intracellular retention during recordings in the superfused chamber, we were able to recognize DCF + cells even after fixation (Fig. 2Ab asterisks). The overlap between DCF and PV fluorescence in the fixed tissue suggested that the occurrence of DCF labeling in PV expressing cells could be even higher than the observed ~50% PV + fraction of the Biocytin filled DCF + cells. To determine the overlap of DCF fluorescence and PV expression, we compared *in situ* DCF labeling with the pattern for PV immunofluorescence in the same slice (Fig. 2B). Following complete reconstruction and counting all cells in 4 slices we found that 94% of the PV cells were also DCF+. The remaining 6% of the PV + DCF negative cells corresponded to neurons located at the lower borders of the tissue block, indicating that they might be false negatives simply out of range in the *in situ* confocal imaging. Remarkably, there were more DCF + than PV + interneurons, indicating that DCF accumulation correlates with the firing properties (i.e. narrow action potentials, non-accommodating cells) but is not dependent on PV expression *per se*.

In order to determine whether DCF-accumulation is a general property of PV + non-accommodating interneurons, we stained acute brain slices from tdTomato parv-Cre transgenic mice (n = 6) with CM-H_2_DCF. Although the fluorescence background in the neuropil was generally higher in acute slices, there was a clear overlap between cytosolic DCF and tdTomato fluorescence (Fig. 2Ca), indicating that H_2_DCF oxidation rates are higher in PV + cells irrespective of the preparation (acute slice vs. slice culture) and the species (rat vs. mice). Patch clamp recordings of DCF + cells in acute rat brain slices again confirmed that DCF + cells were exclusively non-accommodating FS interneurons (Fig. 2Cb, n = 7, inst firing rate: 195 ± 50 Hz, AP halfwidth: 0.67 ± 0.17, n = 7).

### 3.2 Time course and subcellular localization of H_2_DCF oxidation

**Fig. 3:**
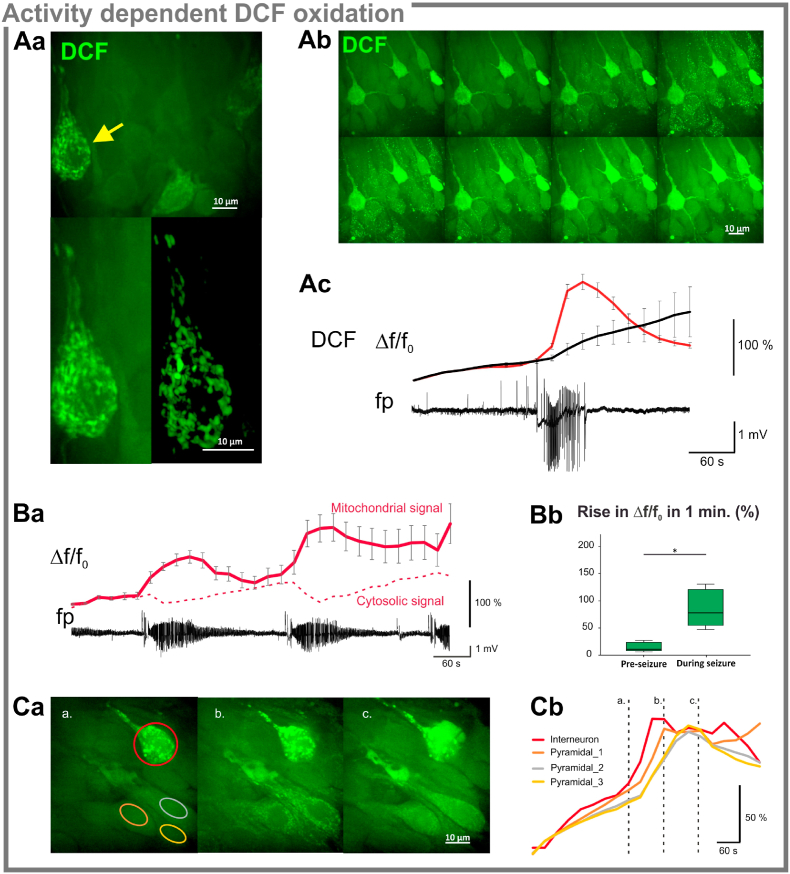
**Kinetics of neuronal activity-dependent H**_**2**_**DCFoxidation** Aa. DCF fluorescence within putative interneurons is not homogeneous. At a higher magnification, filamentous mitochondria-like structures are visible within the cytosol (top). Spatial frequency filtering allows visual differentiation between mitochondrial and cytosolic fluorescence. Bottom pictures represent the 3D-reconstruction of the DCF + cell (yellow arrow), before and after frequency filtering. (Ab) 4AP-induced hypersynchronous events were associated with the sudden appearance of a “dotted” DCF fluorescence pattern in interneurons and principal cells alike (see supplemental time lapse video). (Ac) Frequency filtering revealed that the increase in mitochondrial DCF fluorescence (high spatial frequency component of the signal - red trace) preceded the redistribution of the probe within the cytosol (low spatial frequency component of the signal - black trace). Ba. Recurrent seizure-like events induced repeated oxidation bouts of DCF before the fluorescence reached saturation. Bb. DCF fluorescence baseline continuously rises during the recording due to laser induced oxidation of the probe. However, the slope of the mitochondrial fluorescence significantly increased during seizures as compared to the pre-seizure baseline (*p* = 0.018*,* Wilcoxon signed rank test). Ca. Activity dependent DCF oxidation showed similar kinetics in DCF + interneurons (red trace) and neighbouring principal cells (orange, yellow, grey traces), despite distinct initial fluorescence intensities. (For interpretation of the references to colour in this figure legend, the reader is referred to the Web version of this article.)

As DCF accumulation correlated with the action potential firing pattern, we investigated whether a general increase in neuronal activity would affect the selectivity towards FS interneurons. We monitored the course of H_2_DCF oxidation during spontaneous activity in normal aCSF and following the induction of epileptiform discharges by the application of the voltage-gated potassium channel blocker 4AP [18,22]. Parenchymal DCF fluorescence increased continuously in the recording chamber, likely due to the laser-induced photochemical oxidation of the probe [26]. Thus the steepness of the increase in fluorescence is determined by the dynamic equilibrium between oxidation and the wash out of the probe in favor of the former process. At higher magnification, filamentous organelles were visible in the cytosol of DCF + cells (Fig. 3Aa), whereas the fluorescence in the surrounding pyramidal cells was rather homogeneous, as previously described in [10]. Compared to H_2_DCF, CM-H_2_DCF was less suited for dynamic recordings, as it gave rise to a steadily increasing cytosolic background due to its conjugation with glutathione (GSH).

Application of 4AP induced spontaneous epileptiform activity consisting of short interictal bursts and epochs of recurrent seizure-like events lasting up to 1–3 min. While individual interictal bursts did not affect the overall parenchymal DCF fluorescence, seizure-like events significantly increased the steepness of the slope (Fig. 3AB, *p* = *0.018*). Immediately after the seizure onset, DCF fluorescence appeared in small subcellular spots, followed by a homogeneous increase in the cytosol. Spatial frequency filtering of the image sequence revealed that the fluorescence increase in the high spatial frequency domain -presumably representing mitochondria [23]- preceded the redistribution of the oxidized probe to the cytosol (Fig. 3Abc, supplemental time lapse movie). Remarkably, mitochondrial H_2_DCF oxidation was observed in both pyramidal cells and DCF + interneurons despite the higher initial fluorescence in the latter cell type (Fig. 3C). Thus, the seizure-associated enhancement of cell firing and oxidative energy metabolism resulted in an oxidation of H_2_DCF irrespective of the cell type.

**Fig. 4:**
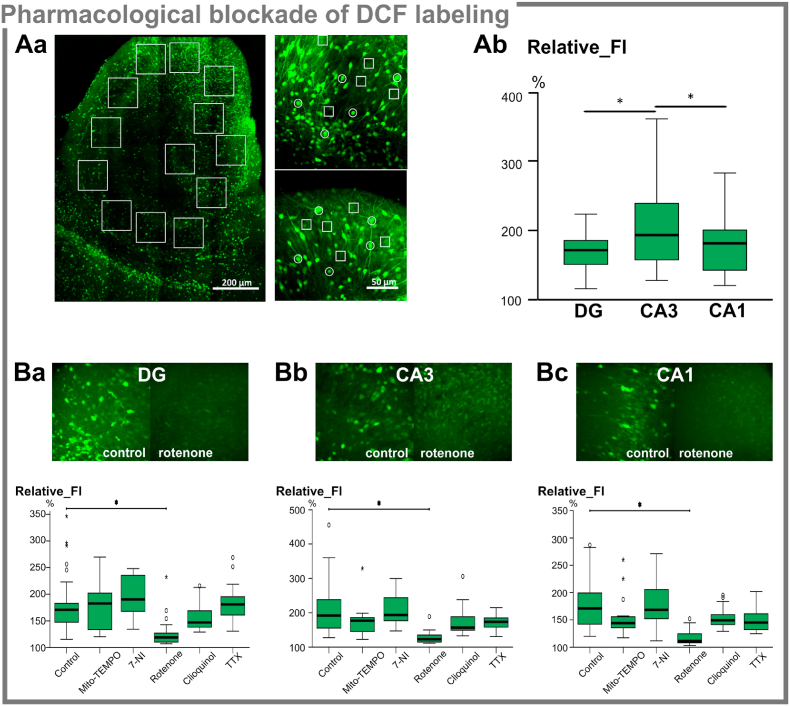
**Pharmacological blockade of DCF labeling in WT cultures** Aa. Left: exemplary image of a slice culture demonstrating the objective fields and ROIs used to evaluate the effects of different blockers and scavengers on the DCF fluorescence pattern. Right: relative fluorescence (relative_Fl) was calculated from parenchymal (square ROIs) and cytosolic (circular ROIs) DCF fluorescence. While the absolute fluorescence depended on staining duration, relative fluorescence did not differ between first and second slice from a membrane (see methods). Ab. Summary graph of the regional differences in relative DCF fluorescence indicating highest intracellular DCF accumulation in the CA3 region (DG *vs.* CA3: *p* = 0.009, CA3 *vs.* CA1 *p* = 0.002, Wilcoxon signed rank test). B. Regional summary of the pharmacological effects on DCF fluorescence patterns in the DG (Ba), CA3 (Bb), and CA1 (Bc). Only the complex I inhibitor rotenone was able to significantly decrease DCF accumulation in all regions (DG: *p* = 0.002, CA3: *p* = 0.0001, CA1: *p* = 0.002, Mood's median test). The pictures represent regional examples from control and rotenone treated slice cultures. The free radical- and free iron scavengers, MitoTempo and Clioquinol as well as the nitric-oxide synthase and voltage dependent sodium channel blockers, 7-nitroindazole and tetrodotoxin did not alter relative fluorescence (see the methods section for details on treatment protocols).

Increased DCF oxidation in pyramidal cells and interneurons during seizure-like events might result from the enhanced electron transport chain activity, the formation of reactive oxygen and nitrogen species, or from the opening of mitochondrial permeability transition pores and release of ferrous iron or cytochrome-c [[27], [28], [29]]. In order to identify the processes underlying the preferential oxidation of DCF in FS interneurons, we randomly assigned 120 slice cultures to control and five pharmacological treatment groups i.e. slice cultures stained in the presence of *i.*, the mitochondrially targeted superoxide-scavenger, MitoTEMPO, *ii*., NO-synthase inhibitor 7NI, *iii*., the iron chelator Clioquinol *iv.*, the electron transport chain complex I inhibitor, rotenone, and *v*., the sodium channel blocker TTX. We determined the relative fluorescence (relative_Fl, fluorescence of DCF + cells relative to surrounding parenchyma) in the regions DG, CA3 and CA1. While absolute values of cellular and parenchymal DCF fluorescence depended on slice rank i.e. staining duration, relative_Fl did not differ within individual treatment groups. In a regional comparison of the control slice cultures, the relative-Fl was significantly higher in the CA3 than CA1 and DG (Friedman test *p* = 0.002 and 0.009 respectively, n = 35, Fig. 4Ab).

While Clioquinol and MitoTEMPO treatments slightly decreased the absolute parenchymal fluorescence, the staining pattern (relative_Fl) did not change significantly (Fig. 4Ba-c). 7NI had no effect, indicating that nitrogen centered radicals did not contribute to the oxidation. By contrast, short term exposure to rotenone prevented intracellular DCF-accumulation and significantly decreased the overall fluorescence, indicating that H_2_DCF oxidation depends on oxidative energy metabolism and intact mitochondrial electron transport (Fig. 4Ba-c).

If H_2_DCF oxidation solely depends on high metabolic rates as a consequence of intensive AP firing, we speculated that the application of the sodium channel blocker TTX would also prevent DCF accumulation in interneurons. However, neither short-term (3 h, not shown) nor long-term (72 h) exposure to TTX (or TTX + CNQX/APV, not shown) was able to reduce the relative fluorescence of DCF + cells over the parenchyma (Fig. 4B. (*p* = 0.002, 0.0001 and 0.002 for the DG, CA3 and CA1 respectively).

## 4 Discussion

The main finding of our study is that the fluorescent probe H_2_DCF (and CM-H_2_DCF) can be used for *in situ* identification of non-accommodating FS interneurons in both acute brain slices and slice cultures. The DCF staining was a characteristic trait of non-accommodating interneurons in general, and strongly correlated with the expression of PV, a marker of FS basket-, chandelier and bitufted cells in particular. The selectivity of the probe towards interneurons correlated with the difference in AP firing as increasing firing rates with 4AP induced a comparable oxidation of the probe in pyramidal cells. On the other hand, blocking action potentials with TTX did not prevent the DCF accumulation in interneurons, indicating that the capacity of interneurons for H_2_DCF oxidation is preserved despite the restricted energy demand. Most remarkably, the oxidation did not depend on the formation of reactive nitrogen or oxygen species or on Fenton-type reactions. Instead, it could be completely abolished by blocking electron transport at the mitochondrial complex I. Along with the oxidation kinetics and subcellular pattern of seizure-dependent fluorescence changes, these results suggest that DCF is oxidized in the mitochondria. Its disproportionate accumulation in non-accomodating interneurons seemingly results from their higher rate of oxidative metabolism and exceptional mitochondrial mass.

Classification of interneurons traditionally relies on electrophysiological and morphological properties as well as on the expression pattern of Ca^2+^-buffer proteins, neuropeptides and transmitter receptors [9]. In addition to this classification, there is a continuum of co-expression profiles of fast-gating ion channels and metabolism-related enzymes across the different interneuron groups, from proximally targeting FS to distal dendrite targeting slow-spiking interneurons [8]. The obvious explanation is that fast ion channel gating kinetics, a prerequisite for firing narrow APs at high frequency, comes at the cost of high ATP demand [2,6]. Indeed, dendritic mitochondrial volume is twice as large in inhibitory interneurons as compared to excitatory dendrites [30], a difference that seems to be maintained between different interneuron classes. Amongst interneurons, FS basket cells have a larger mitochondrial mass than their slow spiking counterparts [7]. On the other hand, PV + FS cells are unable to sustain high firing rates following conditional cytochrome-c oxidase ablation [3]. Thus, the link between channels with fast gating properties, high energy demand and enhanced oxidative capacity has been firmly established [31]. Despite the abundance of transgenic animals carrying fluorescence proteins under different interneuron-specific promoters, it has so far not been possible to label interneurons based on their non-accommodating FS phenotype prior to electrophysiological recordings in brain slice preparations.

Here we present evidence indicating that the conventional fluorescence probe H_2_DCF and its chloromethyl derivative might fill this gap. The tendency for interneuronal accumulation and the co-localization with PV was practically identical for DCF and CM-DCF. Although the conjugation with GSH improved the retention of the probe, it also limited the use of CM-DCF as a dynamic marker for metabolism dependent oxidation.

FS interneurons possess several characteristic traits that might explain the preferential oxidation of the probe. A small percentage of the oxygen entering the electron transport chain is incompletely reduced to superoxide radical, which is then further transformed into the membrane permeable hydrogen peroxide by mitochondrial Mn-superoxide dismutases. Because of their intense oxidative energy metabolism, one might expect abundant superoxide and peroxide formation in FS interneurons. Also, the high mitochondrial mass in these cells is accompanied by high amounts of labile ferrous iron, which is an important catalyst for the initiation and propagation of free radical reactions [32] and might contribute to mitochondrial oxidation of H_2_DCF [15,33]. Nevertheless, in our experimental setting, neither MitoTEMPO nor the iron chelator Clinoquinol was able to decrease DCF oxidation or prevent the dye accumulation in FS interneurons, suggesting that mitochondrial superoxide (or peroxides) are not likely the oxidants of the probe.

Another potential source of peroxynitrite and hydroxyl radicals is the neuronal nitric oxide synthase (NOS), which is highly abundant in certain interneurons such as ivy cells, neurogliaform cells, or a subset of PV, calretinin or somatostatin expressing interneurons [8]. NOS expressing interneurons are preserved in the slice culture preparation and NO contributes to regulation of neuronal activity [29]. However, blocking NO synthesis by 7NI had no effect on the staining pattern, thus ruling out reactions of H_2_DCF with nitrogen-centered radicals.

Only the inhibition of the mitochondrial complex I by rotenone was effective to prevent DCF oxidation and accumulation in interneurons. At 1 μM concentration, rotenone readily blocks gamma oscillations (which rely on FS interneuron function) while leaving stimulus induced synaptic transmission less affected [34]. Interestingly, partial inhibition of complex I is known to increase rather than decrease ROS formation [35]. Thus, if oxidation of H_2_DCF were mediated by ROS, one would expect enhanced DCF labelling in the presence of rotenone. However, in our experiments we found the opposite to be the case. Along with the ineffectiveness of MitoTEMPO at hindering DCF oxidation, these results challenge the hypothesis of direct radical dependent oxidation.

The inhibitory effect of rotenone on H_2_DCF oxidation may be mediated by downstream components of the electron transport chain such as cytochrome-c, which can oxidize H_2_DCF directly or indirectly via a peroxidase-type mechanism [12]. This has been observed in cells undergoing apoptosis where cytochrome-c is released into the cytosol. However, the DCF + interneurons were quite viable and not at all apoptotic - neither in acute slices nor in slice cultures - and continued to display firing rates up to several hundred Hz. Thus the oxidation must take place within intact mitochondria. Further support for this hypothesis comes from a studies showing that oxidation of H_2_DCF is independent of the rate of superoxide formation in vitro, but sensitive to addition of cytochrome-c in the nanomolar range [15,16]. Similarly, another fluorescent probe, the mitochondrially targeted hydroethidine (MitoSox), was described to be oxidized by cytochrome-c [36]. Considering that larger mitochondrial mass (and consequently larger cytochrome-c levels) correlate with H_2_DCF oxidation, this would also explain the apparent controversy that oxidation is enhanced by neuronal activity (as seen in 4AP-treated slice cultures) but even several days of complete block of action potentials and synaptic transmission could not prevent the disproportionate accumulation of DCF in FS interneurons. Thus, the difference in baseline metabolic activity between principal cells and FS interneurons seem to be independent of the actual AP firing, but any additional enhancement of oxidative metabolism will inevitably increase H_2_DCF oxidation.

In conclusion, FS interneurons such as PV expressing basket cells are fundamental in orchestrating network activity, especially in the high frequency range [37]. Indeed, PV-Cox10 conditional knockout animals display abnormal gamma oscillations and social behavioural deficits. Consequently defective mitochondria, impaired antioxidant systems and dysfunctional FS interneurons have been implicated in multiple neuropsychiatric diseases such as schizophrenia or depression. We hope our findings will open new avenues for neuronal network analysis based on multipatch-recordings by facilitating identification of non-accommodating FS interneurons in the brain slice preparation [38].

## Declaration of competing interest

The authors declare no competing financial or non-financial interests.
