# Supplementary material for: In situ labeling of non-accommodating interneurons based on metabolic rates
Source: Redox Biol. 2020 Nov 28;38:101798. doi: 10.1016/j.redox.2020.101798 (PMC7724199; doi:10.1016/j.redox.2020.101798)
Supplement: Multimedia component 2 [file mmc2.docx]

**Supplemental Methods**

*Preparation of brain slices and slice cultures*

Slice cultures were prepared and maintained according to the Stoppini method. Hippocampi from either wild type or VGAT-YFP transgenic rat pups of either sex were extracted on postnatal day 6-7. The isolated hippocampus was cut into 400 µm slices perpendicular to the dorsoventral axis with a McIllvain Tissue Chopper. Two slices were positioned on cell culture membrane inserts (Millicell-CM, Millipore) and maintained in six-well plates filled with 1 mL culture medium (50% MEM, 25% HBSS, 25% Horse Serum and 1 mM L-glutamine, pH set to 7.3) in a humidified CO_2_ incubator (5% CO_2_). Animals of either sex were used indiscriminately as we did not observe any difference in the staining pattern in our previous study ([^10^](#5lr505gmykvu)). As maturation of the neuronal network and antioxidant systems occurs in the absence of gonadal sex hormones, culture conditions have the larger impact on the network and antioxidant capacity than the sex of the animals ([^18^](#nr0touiuygat)).

Acute slices were prepared from anesthetized (2% isoflurane) tdTomato parv-Cre transgenic mice (> P50, derived from crossing B6.Cg-Gt(ROSA)26Sortm9(CAG-tdTomato)Hze/J and B6;129P2-Pvalbtm1(cre)Arbr/J strains) or young adult (P27-29) Wistar rats. Following decapitation brains were rapidly removed and submerged in ice-cold sucrose-based and carbogenated (95% O_2_/ 5% CO_2_) slicing solution containing in mM: 80 NaCl, 2.5 KCl, 3 MgCl_2_, 0.5 CaCl_2_, 25 glucose, 85 sucrose, 1.25 NaH_2_PO_4_ and 25 NaHCO_3_ (320–330 mOsm). The brain was cut into 300 µm thick horizontal slices with a vibratome (Leica VT1200 S, Wetzlar, Germany) containing the hippocampus and entorhinal cortex. Slices were subsequently stored in the sucrose aCSF solution heated to 34 °C for ~30 min followed by storage at room temperature. We never observed sex dependent differences in the staining pattern of acute slices.

*Electrophysiology*

Acute rat brain slices or slice cultures (DIV 5-14) were transferred to a recording chamber perfused with warmed (30-32 °C) carbogen saturated (95 % CO_2_, 5 % O_2_) artificial cerebrospinal fluid (aCSF, in mM: NaCl 129, KCl 3, NaH_2_PO_4_ 1.25, MgSO_4_ 1.8, CaCl_2_ 1.6, NaHCO_3_ 26, and glucose 10, pH 7.3). A NoranOz laser scanning confocal microscope (Prairie Technologies), equipped with a 60x (N.A. 0.9) water-immersion objective was used to identify fluorescent target cells. Whole cell recordings were obtained in YFP+ interneurons (VGAT-YFP rats) or in DCF+ cells (WT rats) throughout the cornu ammonis by using pipette solutions (K-gluconate, 135, HEPES 10, KCL 6, MgCl_2_ 2, EGTA 0.2, Na_2_ATP 2, Na_2_GTP 0.5, Na_2_-Phosphocreatine 5, Biocytin 0.1%, or K-gluconate 135; NaCl 4; CaCl_2_ 0,05; HEPES 10; EGTA 1; Mg-ATP 2, 270-290 mOsm, pH 7.3) and by using the amplifiers MultiClamp 700B (Axon CNS, Molecular Devices, Sunnyvale, CA, USA) or Pico2 (Tecella, Foothill Ranch, CA, USA). Data were filtered at 3 kHz, digitized at 10 kHz (Digidata 1440A, Axon CNS, Molecular Devices, Sunnyvale, CA, USA) and recorded by using the software Clampex 10 (Molecular Devices). Zero net current membrane potential (Em), membrane capacity (Cm), membrane resistance (Rm) and access resistance (Ra), were determined automatically in voltage clamp (VC) immediately after breakthrough. Recordings with unreliable Em (holding current > 100 pA to maintain Em in CC) and/or Rm < 3*Ra were not included in the evaluation. One-minute long gap-free traces were recorded in VC for analysis of synaptic input and presence of spikelets. In current clamp mode (CC) bridge balance was automatically compensated before recording 1 s long hyperpolarizing and depolarizing steps of 25 mV to evoke AP-trains.

*Immunohistochemistry*

Slice cultures were fixed with 4 % paraformaldehyde / 4 % sucrose in phosphate-buffered saline (PBS) 0.1 M overnight at 4 °C and stored in 30 % sucrose / PBS. For immunofluorescent labelling, slices were carefully detached from the PTFE membranes and processed free-floating. Incubation with a mouse derived anti-parvalbumin antibody (1:1000; Millipore) in 0.1 % Triton X-100 / PBS for 3 nights was followed by incubation for 24 hours with a goat anti-mouse Cy3 secondary antibody (1:100; Millipore) and avidin-conjugated Alexa Fluor488 (1:1000, Invitrogen). Slices were mounted on adhesion slides (Superfrost Plus, Fisher Scientific) and coverslipped with Fluoromount-G, (SouthernBiotech). To minimize fixed tissue compression we used an imaging spacer (120 µm, Grace Bio-Labs SecureSeal™).

*Pharmacology*

To identify possible target mechanism responsible for H_2_DCF oxidation, slice cultures were treated prior to and during the staining procedure with the following substances: the mitochondrial superoxide-scavenger (2-(2,2,6,6-tetramethylpiperidin-1-oxyl-4-ylamino) -2-oxoethyl) triphenylphosphonium chloride (MitoTEMPO, Santa Cruz Biotechnology, Inc, Dallas, TX, USA); the electron transport chain complex I inhibitor rotenone (Sigma-Aldrich, St. Louis, MO, USA); Neuronal NO-synthase inhibitor 7-nitroindazole (7-NI, Sigma-Aldrich, St. Louis, MO, USA); the iron chelator 5-Chloro-7-iodo-8-quinolinol (Clioquinol, BioVision, Milpitas, CA, USA) as well as the voltage gated sodium channel inhibitor tetrodotoxin (TTX, BIOTREND Chemicals AG, Cologne, Germany). In a subset of TTX exposed slices the glutamate receptor inhibitors 6-cyano-7-nitroquinoxaline-2,3-dione (CNQX), (2R)-amino-5-phosphonopentanoate (DL-APV, 50 µM each) were also added to the medium. Mito-TEMPO was solved in 1 ml serum-free culture medium, stock solutions of 7-NI, rotenone, clioquinol were prepared in DMSO (final concentration 0.1%), while TTX was prepared in an acidic buffer solution (pH=4.8). Table 2 summarizes the treatment protocol for each group. For exposure longer than two hours the substances were added to the horse-serum-containing culture medium, followed by the staining in serum-free culture medium containing the same concentration of the drugs in addition to CM-H_2_DCF-DA. To limit the potentially toxic effects of rotenone and Clioquinol, these exposures were kept short and applied simultaneously with the staining. Epileptiform activity was induced by adding 4AP (100 µM, Bio-Techne GmbH, Wiesbaden-Nordenstadt, Germany) to the aCSF.

*Image acquisition and evaluation*

Fluorescence recordings of the kinetics and pharmacology of H_2_DCF oxidation were obtained with a spinning disk confocal microscope (Andor Revolution, BFIOptilas GmbH, Gröbenzell, Germany), equipped with an EMCCD camera (Andor iXonEM+). Slice cultures were incubated at least ~30 min before image acquisition with the membrane permeable probes H_2_DCF-DA or CM-H_2_DCF-DA (20 µM), for kinetics and pharmacology, respectively. The slices from an individual culture membrane were imaged subsequently, resulting in an approximately 30-minute long difference in treatment/staining duration between slices based on their rank, i.e. first, second (and third). Following staining in the presence of the blockers (see above), 8-15 Z-stacks were obtained covering all the regions of the hippocampus i.e. the dentate gyrus, CA3 and CA1. Imaging parameters and laser intensity were constant throughout all sessions. ROIs of 5 representative cells and arbitrary selected areas from the parenchym were placed on the maximum intensity projection of the image stacks (Figure 4Aa) as a marker of cytosolic DCF accumulation. While the absolute fluorescence depended on the duration of the staining, hence on the rank of a slice from a culture membrane, the relative fluorescence was unaffected from staining length, showing no significant differences within each individual treatment group.

To record the activity-dependent oxidation of the probe, z-stacks (10-15 focal planes, 1-1.2 µm distance) were obtained every 20-30 s over the CA3, while simultaneously recording the local field potentials of 4AP-induced network activity. The short exposition (100 ms) and the low sampling frequency were chosen to limit the influence of the light-induced auto-oxidation of the probe. Putative mitochondrial components of the DCF fluorescence were isolated by using spatial frequency filtering plugins of the ImageJ distribution FIJI (fiji.sc), whereas cytosolic fluorescence changes were measured over apparently mitochondria-free cytosolic regions (nucleus). In brief, following transformation into the spatial frequency domain, the images were multiplied with an appropriate low pass-filter function and converted back to the spatial domain with reverse FFT. The cut-off frequency of the filter function was selected by iterating the process with increasing spatial frequency filters and comparing fluorescence images originating from mitochondria and nucleus. Changes in the DCF fluorescence intensity are presented as Δf/f_0_ over time, f_0_ representing the average fluorescence of the first 1 min of the recording.

DCF and PV quantification was conducted in panorama images of the entire hippocampal slice, obtained by using the large scan function provided with the Nikon’s A1R multiphoton confocal microscope (25× N.A. 1.1 objective, Nikon, Shinagawa, Tokyo, Japan) at the AMBIO Life Cell Imaging Core Facility (AMBIO.charite.de). The same function was used to image the cultures after immunohistochemistry for PV (InSight DeepSee pulsed TiSa laser (Spectra-Physics, Santa Clara, CA, US), laser lines 960 nm and 1040 nm, for DCF and Cy3 respectively. The distance between focal planes was kept constant (1.2 µm) while the number of z stacks acquired varied depending on the thickness of the slices. To overcome the shrinkage and distortion of the fixed tissue we used a hallmark-based image registration procedure (ImageJ plugin BigWarp; and the CellCounter plugin to determine the percentage of DCF + PV + cells.

*Statistics*

Statistical analysis was conducted in SPSS software package (IBM, SPSS 24). Recordings from YFP and WT cultures aged DIV 5-14 were used for statistical analysis of patch clamp experiments (205 cells recorded, YFP = 122; WT = 83). Normality of distribution of the electrophysiological variables was tested for WT and YFP cases independently by analyzing histograms, Q-Q Plots and de-trended normal Q-Q Plots via the Shapiro-Wilk test and median values compared using Mood´s median test. We used binary logistic regression to explore how informative the DCF labeling is in predicting the FS firing pattern. First, we fit the model hierarchically entering potential predictor variables and subsequently excluded those that did not significantly contribute to the model (fit), including only the explanatory predictor variables YFP/WT, AP halfwidth, membrane capacity, access resistance, relative interspike interval (reported in supplemental Table). These variables improved the prediction of membership to the FS group from 66,8 % correct in the baseline model (before input of predictor variables) to 92,0 % correct in the final model.

The assumption of linearity of the logit was not violated as the interaction terms consisting of each predictor multiplied by its natural logarithm all had significance values greater than 0,05. We did not find multicollinearity among the predictor variables (collinearity statistics: all the tolerance values were greater than 0,1 and all variance inflation factor values were smaller than 10). Parameters of model assessment indicated a good model fit (-2LLbaseline = 237,59 ; -2LLnew = 82,37 ; likelihood ratio (χ2) = (-2LLbaseline) – (-2LLnew) = 155,22 (p < 0,001) ; R2 = 0,564 (Cox & Snell), 0,784 (Nagelkerke).

Because the 95% CI for the odds ratio (change in odds after unit increase in the predictor) was greater than 1 for the variables YFP/WT and relative interspike interval, the odds of the outcome FS for the variable firing pattern (with FS cells set as the reference category) increased with these variables. Thus, the DCF + WT cells and neurons with large interspike interval ratios are associated with the FS firing pattern. The contrary holds true for the predictor variables AP halfwidth and membrane capacity as both have odds ratios smaller than 1, indicating an association of shorter AP half widths and lower membrane capacities with the FS firing pattern.

The DCF fluorescence data obtained in different treatment groups was not normally distributed (Shapiro-Wilk). Hence, we used the nonparametric median test to compare the effect of the different treatments on the oxidation of H_2_DCF. The significance values of the post-hoc test for pairwise comparison between the groups was adjusted by the Bonferroni correction for multiple tests.

The nonparametric median test showed significant differences in the absolute values of cellular and parenchymal DCF fluorescence but not the relative fluorescence values when comparing slices based on their rank i.e. staining duration. The nonparametric Friedman test was used to compare the relative fluorescence values between the three different areas of the hippocampus, i.e. the DG, CA1, and CA3, in the control group. For pairwise comparison Wilcoxon signed-rank test and Sign test were used with Bonferroni adjustment. Wilcoxon signed-rank test was also used to compare the rise in the fluorescent signal within one minute before and during seizures.

**Supplemental Table**

Summary of the electrophysiological parameters used for characterization of the recorded interneurons in WT and VGAT_YFP slice cultures. Median and first and third quartile values for the groups accommodating (n=66) and fast spiking non-accommodating (FS, n=139) neurons are presented based on the classification via logistic regression.

| **Parameters** | | **Phenotype** | **n** | **Q1** | **Q2** | **Q3** |
| --- | --- | --- | --- | --- | --- | --- |
| passive properties | resting membrane potential E0 (mV) | accomodating | 66 | -58,00 | -54,00 | -51,00 |
|  |  | FS | 139 | -58,00 | -54,00 | -51,00 |
|  | membrane capacity Cm (pF) | accomodating | 66 | 55,52 | 87,81 | 122,62 |
|  |  | FS | 139 | 76,56 | 111,32 | 150,09 |
|  | membrane resistance Rm (MOhm) | accomodating | 66 | 127,05 | 163,36 | 303,11 |
|  |  | FS | 139 | 82,09 | 128,28 | 179,24 |
|  | Series resistance Rs (MOhm) | accomodating | 66 | 8,43 | 13,34 | 20,23 |
|  |  | FS | 139 | 9,49 | 15,26 | 24,23 |
| active properties during 1 s depolarizing step | AP frequency (Hz) | accomodating | 65 | 45,82 | 55,33 | 60,84 |
|  |  | FS | 135 | 64,73 | 83,33 | 132,45 |
|  | AP halfwidth (ms) | accomodating | 62 | 1,17 | 1,34 | 1,44 |
|  |  | FS | 125 | 0,63 | 0,89 | 1,18 |
|  | AP amplitude (mV) | accomodating | 65 | 38,35 | 48,27 | 56,31 |
|  |  | FS | 135 | 44,52 | 51,32 | 58,85 |
|  | A1: frequency of first 5 AP (Hz) | accomodating | 65 | 61,49 | 76,93 | 92,04 |
|  |  | FS | 135 | 66,15 | 85,51 | 108,85 |
|  | B1: interspike interval of first 5 AP (ms) | accomodating | 65 | 10,98 | 13,55 | 16,92 |
|  |  | FS | 135 | 9,20 | 12,00 | 15,30 |
|  | A2: frequency of last 5 AP (Hz) | accomodating | 65 | 33,95 | 42,46 | 51,57 |
|  |  | FS | 135 | 59,39 | 77,41 | 123,08 |
|  | B2: interspike interval of last 5 AP (ms) | accomodating | 65 | 20,00 | 24,68 | 31,65 |
|  |  | FS | 135 | 8,38 | 13,10 | 17,03 |
|  | hw (ms) of second to last AP during 1 s train 150 pA step | accomodating | 65 | 1,32 | 1,87 | 2,26 |
|  |  | FS | 135 | 0,84 | 0,99 | 1,17 |
| computed | relative frequency (A1 : A2) | accomodating | 65 | 0,43 | 0,54 | 0,75 |
|  |  | FS | 135 | 0,86 | 1,01 | 1,18 |
|  | relative interspike interval (B1 : B2) | accomodating | 57 | 1,26 | 1,60 | 1,90 |
|  |  | FS | 120 | 0,65 | 0,86 | 1,45 |
